# Paclitaxel-coated balloon catheter for benign esophageal stenosis in a rabbit model

**DOI:** 10.1038/s41598-024-53078-0

**Published:** 2024-01-31

**Authors:** Kewei Ren, Jianan Wang, Yahua Li, Zongming Li, Zihe Zhou, Kunpeng Wu, Yifan Li, Xiaoyong Ge, Jianzhuang Ren, Xinwei Han

**Affiliations:** 1 Department of Interventional Radiology, The First Affiliated Hospital of Zhengzhou University, Zhengzhou, 450052 Henan People’s Republic of China; 2 Engineering Technology Research Center for Minimally Invasive, Interventional Tumors of Henan Province, Zhengzhou, Henan 450052 People’s Republic of China; 3 Department of Interventional Radiotherapy, Zhoukou Center Hospital, Zhoukou, 46000 People’s Republic of China; 4 Interventional Treatment and Clinical Research Center of Henan Province, Zhengzhou, 450052 People’s Republic of China; 5 Interventional Institute of Zhengzhou University, Zhengzhou, 450052 People’s Republic of China

**Keywords:** Gastrointestinal models, Oesophagogastroscopy

## Abstract

Most patients with benign esophageal stenosis require multiple or even continuous balloon dilation treatments to achieve symptom relief. In this study, eighteen rabbits were used to establish an esophageal benign stenosis model and were divided into a control group (n = 6), a balloon group (n = 6) and a PTX-coated balloon group (n = 6) to evaluate the feasibility and effectiveness of paclitaxel (PTX)-coated balloons for the rabbit esophageal benign stenosis model. The weight and esophageal diameter were recorded every 2 weeks until 8 weeks post-surgery. Hematoxylin–eosin staining, Masson’s trichrome staining and immunohistochemical staining were performed for pathological analysis. Four weeks post-operation, there was a significant difference in weight between the control group and the balloon group (p = 0.01) and between the control group and the PTX balloon group (p = 0.01). There was a significant difference in the esophageal diameter between the balloon group and the PTX balloon group at 8 weeks post-operation (p = 0.02). Four weeks post-operation, the degree of inflammatory cell infiltration in the PTX balloon group was significantly lower than that in the control group (p = 0.002) and balloon group (p = 0.001). The degree of collagen deposition in the PTX balloon group was significantly lower than that in the control group (p = 0.002) and balloon group (p = 0.03). Eight weeks post-operation, the percentage of cells positive for TGF-β (p < 0.001), the degree of inflammatory cell infiltration (p = 0.02) and the degree of collagen deposition (p = 0.02) in the PTX balloon group were significantly lower than those in the balloon group. Therefore, PTX-coated balloons may alleviate the local inflammatory response and collagen deposition when used during dilation treatment of benign esophageal stenosis.

## Introduction

Esophageal stenosis, which is a narrowing of the esophageal lumen caused by various diseases, has an estimated incidence of 1.1/100,000 people per year^1^. The causes of esophageal stenosis can be divided into benign and malignant. The incidence of malignant stenosis is greater than that of benign stenosis.

There are three main causes of benign esophageal stenosis: endogenous esophageal diseases, iatrogenic esophageal injuries and accidents. Endogenous esophageal diseases include digestive esophagitis, eosinophilic esophagitis, and other diseases of the squamous epithelium. Iatrogenic esophageal injuries include endoscopic submucosal dissection (ESD) of large esophageal lesions and anastomotic stenosis after surgery, radiofrequency ablation and thoracic radiation therapy. Accidents include the accidental ingestion of chemicals and corrosive items^2–4^^.^ The clinical manifestations of benign esophageal stenosis are mainly intractable dysphagia. In addition, some patients experience nausea, vomiting, acid reflux, a burning sensation behind the sternum, and aspiration pneumonia, which greatly affects their quality of life^5,6^.

The purpose of treating benign esophageal stenosis is to reduce the symptoms of feeding difficulties in patients, restore normal feeding and water intake, improve the quality of life of patients, and avoid the occurrence of strict esophageal-related complications such as malnutrition and the recurrence of benign esophageal stenosis. Balloon dilatation is the preferred treatment for benign esophageal stenosis and has the advantages of high safety and a low incidence of serious complications^7^. Most patients with benign esophageal stenosis do not achieve symptom relief through one dilation, and some patients need multiple or even continuous dilation treatments to achieve symptom relief. Compared with simple benign esophageal stenosis, complex esophageal stenosis is more difficult to treat, requiring more dilation attempts and resulting in greater complications and recurrence rates of stenosis than simple benign esophageal stenosis^8,9^. Previous reports have shown that the complication probability of esophageal perforation caused by balloon dilation is between 0.1 and 0.4%, and the complication probability of esophageal perforation caused by penetrating band dilation is between 0.1 and 5%^10^.

Low-dose paclitaxel (PTX) can inhibit the proliferation and migration of fibroblasts by inhibiting the transforming growth factor-β1/Smad3 pathway. The widespread application of drug-coated balloons (DCBs) in the treatment of coronary artery and peripheral artery stenosis provides a new way of thinking about the treatment of benign esophageal stenosis and its associated relapses^11,12^. PTX may be applied locally to treat esophageal stenosis and inhibit granulation tissue proliferation. In this study, a PTX balloon catheter was applied in a rabbit model of benign esophageal stenosis to evaluate its feasibility and effectiveness.

## Methods

This study is reported in accordance with the ARRIVE guidelines. Xylazine hydrochloride (1 mg/kg) combined with pentobarbital sodium (0.5 mg/kg) was intramuscularly injected for anesthesia. Eighteen New Zealand rabbits (both male and female) (weight: 3.0 ± 0.2 kg) were evenly divided into a control group (n = 6), a balloon group (n = 6) and a PTX-coated balloon group (n = 6). The PTX-coating ballons were commercially available (Zylox Medical Device Co., Ltd.). All the rabbits were fed in a single cage. The ambient temperature in the room was 22 ± 2 °C, and the relative humidity was 45 ± 15% with a 12-h light/dark cycle. Standard food and water were accessible freely. The rabbits were fasted for 12 h before the operation, and water was forbidden for 4 h. The weight was recorded before the operation. This animal study was approved by the Committee on the Ethics of Animal Experiments of Zhengzhou University (approval number: 20201011). Animal studies were conducted at the Henan Key Laboratory for Pharmacology of Liver Disease.

### Establishment of an esophageal benign stenosis model

**Figure 1:**
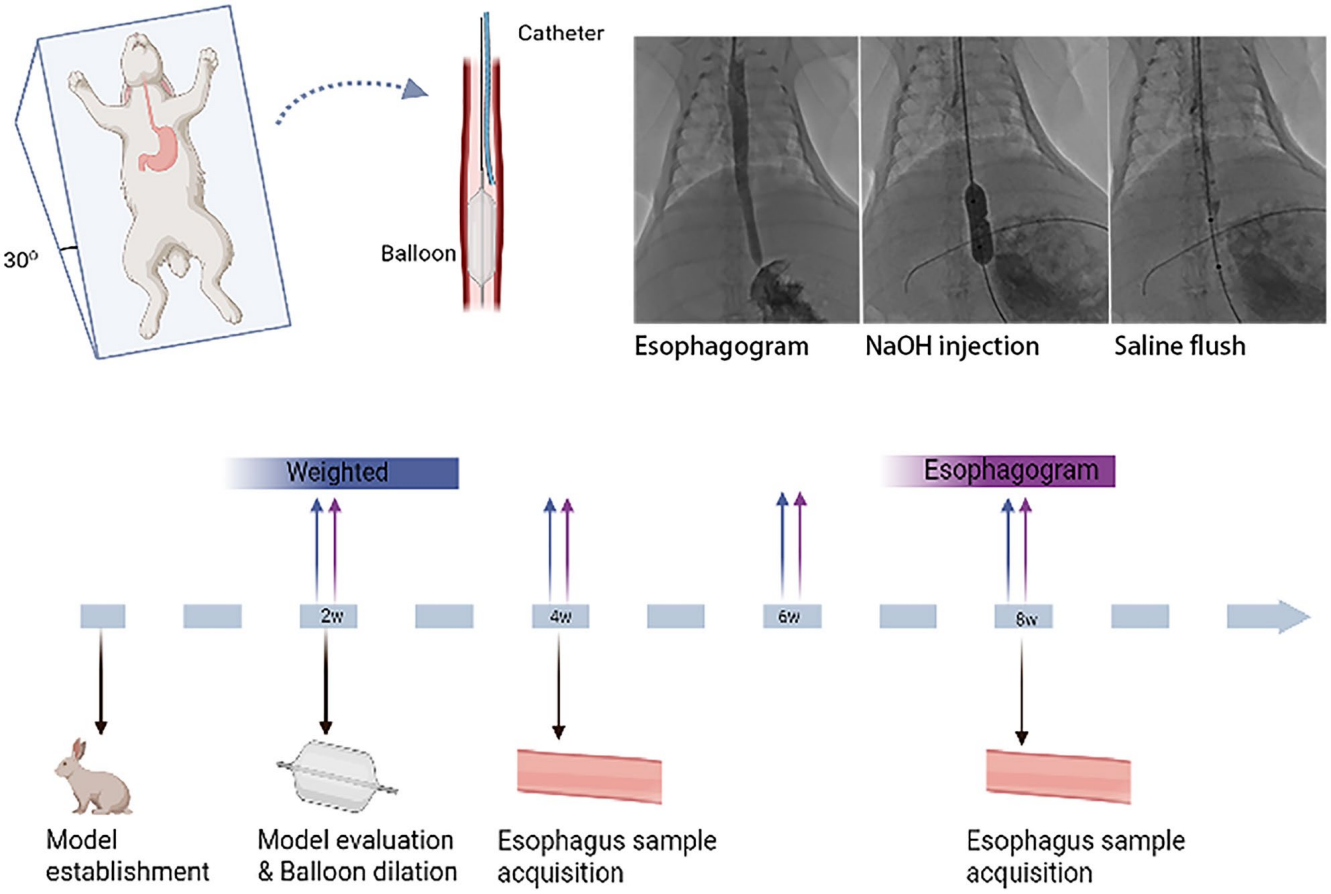
Diagram of the establishment of an esophageal benign stenosis model under the guidance of fluoroscopy and the flow chart of this study.

After anesthesia, the head of the rabbit was fixed at a 30° elevation. A guidewire with a 5F catheter and 9F sheath was introduced to the esophagus via the mouth. The catheter was adjusted to the stomach, and the guidewire was withdrawn. An esophagogram was taken after the catheter was withdrawn and the contrast agent was injected. The diameter of the esophagus was measured and recorded. A balloon with a maximum diameter of 20 mm was introduced approximately 2 cm above the cardia. A catheter with tip-side holes was placed above the balloon. After the balloon expanded, 1 mL of 4% NaOH was injected, and the mixture was kept for 30 s. Then, the balloon was introduced to the stomach. Ten milliliters of saline was injected slowly through the catheter to flush the esophageal lumen. After saline injection, the catheter, guidewire and balloon catheter were removed. The procedure for establishing the esophageal benign stenosis model was completed (Fig. 1). Ceftiofuran sodium was injected intramuscularly at a dosage of 3 mg/kg for the following 3 days.

### Esophagography and balloon dilation

**Figure 2:**
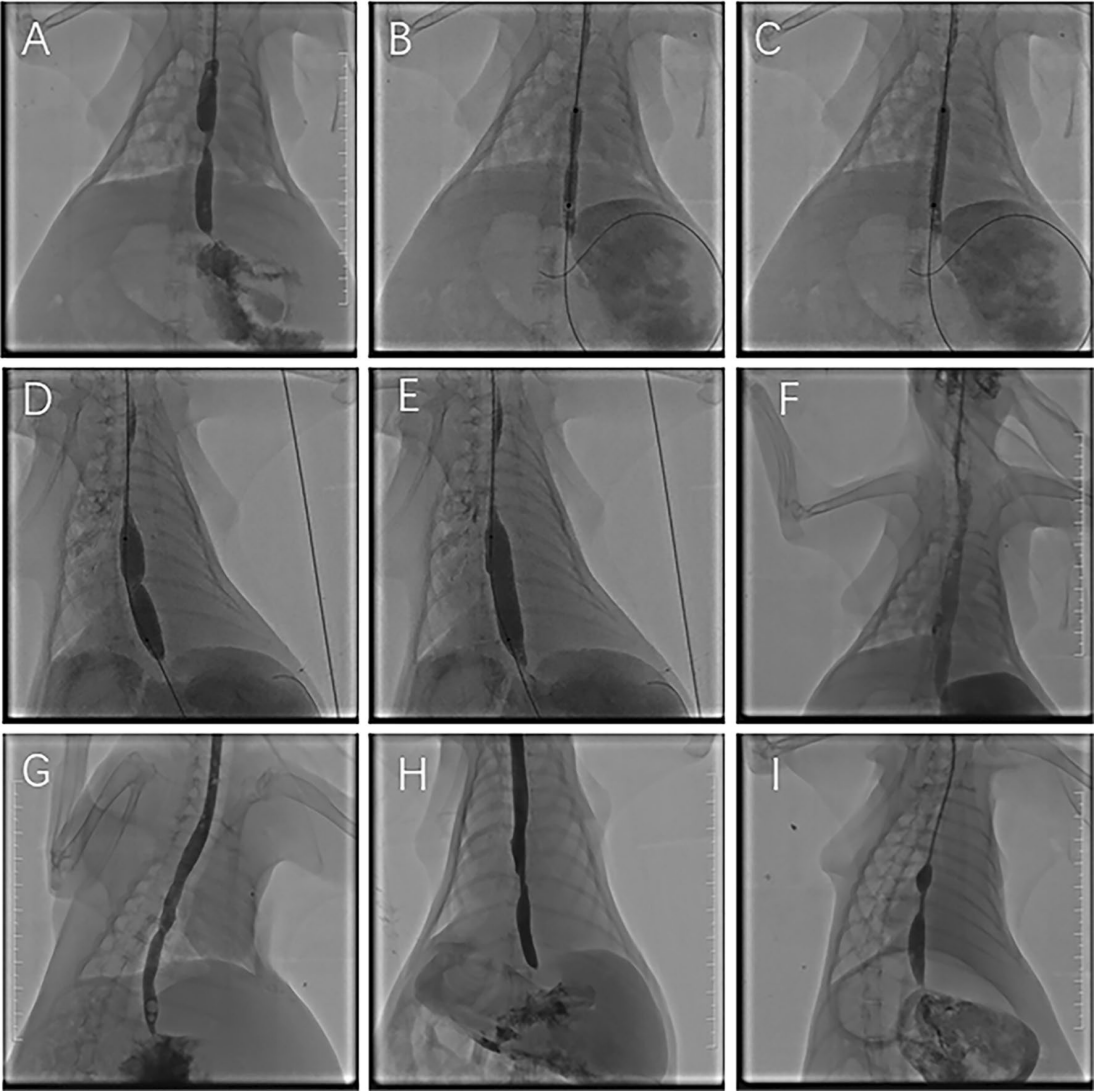
Representative images from the balloon group. (**A**) Two weeks after the operation, an esophagogram showed esophageal stenosis. (**B**) Balloon dilation at the stenosis location with a diameter of 6 mm. (**C**) The diameter of the 6 mm balloon was completely dilated. (**D**) Balloon dilation at the stenosis location with a diameter of 10 mm. (**E**) The diameter of the 10 mm balloon was completely dilated. (**F**) Esophagography after dilation showed that the esophageal lumen stenosis was enlarged, and the contrast agent was passed smoothly. (**G**) At 4 w post-operation (after dilation for 2 w), an esophagogram showed patency of the esophageal lumen. (**H**) At 6 w post-operation (after dilation for 4 w), an esophagogram showed that the diameter of the esophageal lumen had decreased. (**I**) At 8 w post-operation (after dilation for 6 w), an esophagogram showed the recurrence of stenosis.

**Figure 3:**
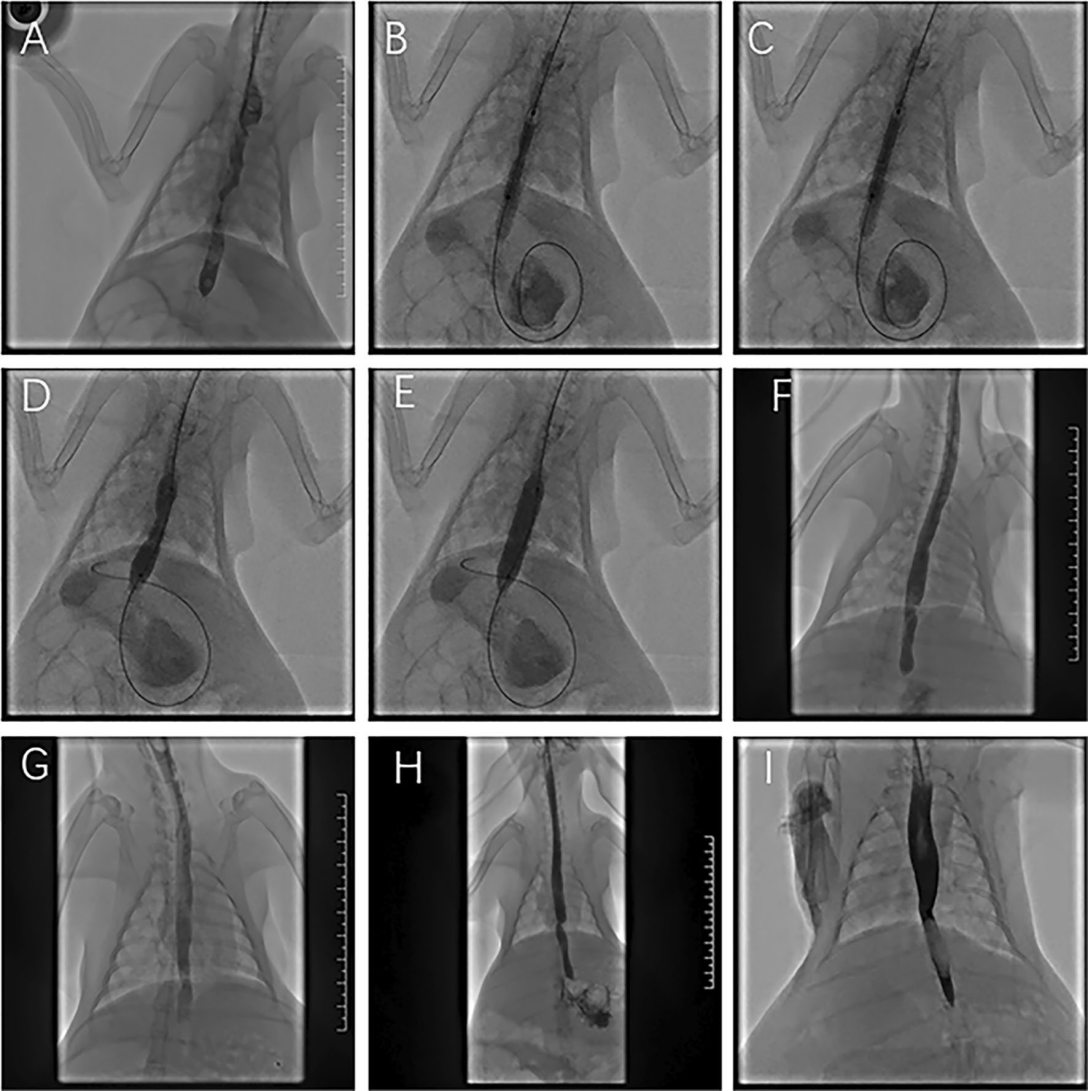
Representative images of the PTX-coated balloon group. (**A**) Two weeks after the operation, an esophagogram showed esophageal stenosis. (**B**) PTX-coated balloon dilation at the stenosis location with a diameter of 6 mm. (**C**) The diameter of the 6 mm balloon was completely dilated. (**D**) Balloon dilation at the stenosis location with a diameter of 10 mm. (**E**) The diameter of the 10 mm balloon was completely dilated. (**F**) Esophagography after dilation showed that the esophageal lumen stenosis was enlarged, and the contrast agent was passed smoothly. (**G**) At 4 w post-operation (after dilation for 2 w), an esophagogram showed patency of the esophageal lumen. (**H**) At 6 w post-operation (after dilation for 4 w), an esophagogram showed that the diameter of the esophageal lumen had decreased. (**I**) At 8 w post-operation (after dilation for 6 w), esophagography showed that the diameter of the esophageal lumen was slightly decreased.

After the establishment of the esophageal benign stenosis model for 2 weeks, esophagography was performed to measure the diameter of the stenotic esophagus. In the control group, only esophagography was performed. In the balloon group and PTX balloon group, a balloon 6 mm in diameter with or without PTX coating was utilized to dilate the stenotic esophagus first. Then, a balloon 10 mm in diameter was used for dilation. Finally, esophagography was performed again to evaluate whether complications occurred (Figs. 2 and 3). Ceftiofuran sodium was applied for the following 3 days.

### Follow-up

During the follow-up, the rabbits in the 3 groups were weighed, and esophagograms were taken to measure the diameter of the stenotic esophagus every 2 weeks until 8 weeks post-model establishment. The rabbits were sacrificed after esophagography at 4 and 8 weeks after model establishment. The esophagus was fixed in 10% neutral buffered formalin for 24 h.

### Histological analysis

Paraffin blocks with the esophagus were cut into 5-μm-thick sections to obtain slides. Hematoxylin–eosin (HE) staining Masson’s trichrome (MTS) and immunohistochemical (IHC) staining protocols were used for pathological analysis. The degree of inflammatory cell infiltration was determined subjectively according to the distribution and density of inflammatory cells.

When single leukocyte infiltration was occasionally observed, it was evaluated as mild and scored as 0. When the leukocytes had patchy infiltration, they were judged as mild to moderate and scored a (1) When leukocytes were aggregated and individuals could not be distinguished, they were regarded as moderate and scored as (2) When there was diffuse infiltration of leukocytes throughout the submucosal layer, a moderate to severe degree was documented and was scored as (3) When there was diffuse infiltration with multiple necrotic foci, the severity of the lesion was determined, and a score of 4 was given. Collagen deposition was also subjectively determined, where 0 indicated mild deposition; (1) mild to moderate deposition; (2) moderate deposition; (3) moderate to severe deposition; and (4) severe deposition. ImageJ software was used to analyze the IHC results.

### Statistical analysis

Figures were generated with GraphPad Prism 9 software. Differences between groups were analyzed by analysis of variance. Post hoc comparisons were performed using the Bonferroni method. Fisher’s exact test and the chi-square test were used to compare categorical variables between groups. The statistical analysis was performed using SPSS 21.0.

### Ethics approval and consent to participate

This animal study was approved by the Committee on the Ethics of Animal Experiments of Zhengzhou University—approval: 20201011. All methods were performed in accordance with the relevant guidelines and regulations. Animal studies were conducted at Henan Key Laboratory for Pharmacology of Liver Disease.

## Results

Among the 18 rabbits, 17 had a successfully established benign esophageal stenosis model. Only 1 rabbit died 6 days post-operation. The success rate was 94.4%. During the balloon dilation procedure, one rabbit developed an esophageal fistula and died on the second day. Two rabbits were added for the benign esophageal stenosis model established to perform balloon dilation. Three rabbits in the control group were sacrificed 2 weeks post-operation. The remaining 3 rabbits in the control group died within the next 2 weeks. The weight of the rabbits at the time of death was regarded as the weight of the rabbits in the control group at 4 weeks post-operation. The esophagogram diameter data could not be obtained.

### Weight changes

**Figure 4:**
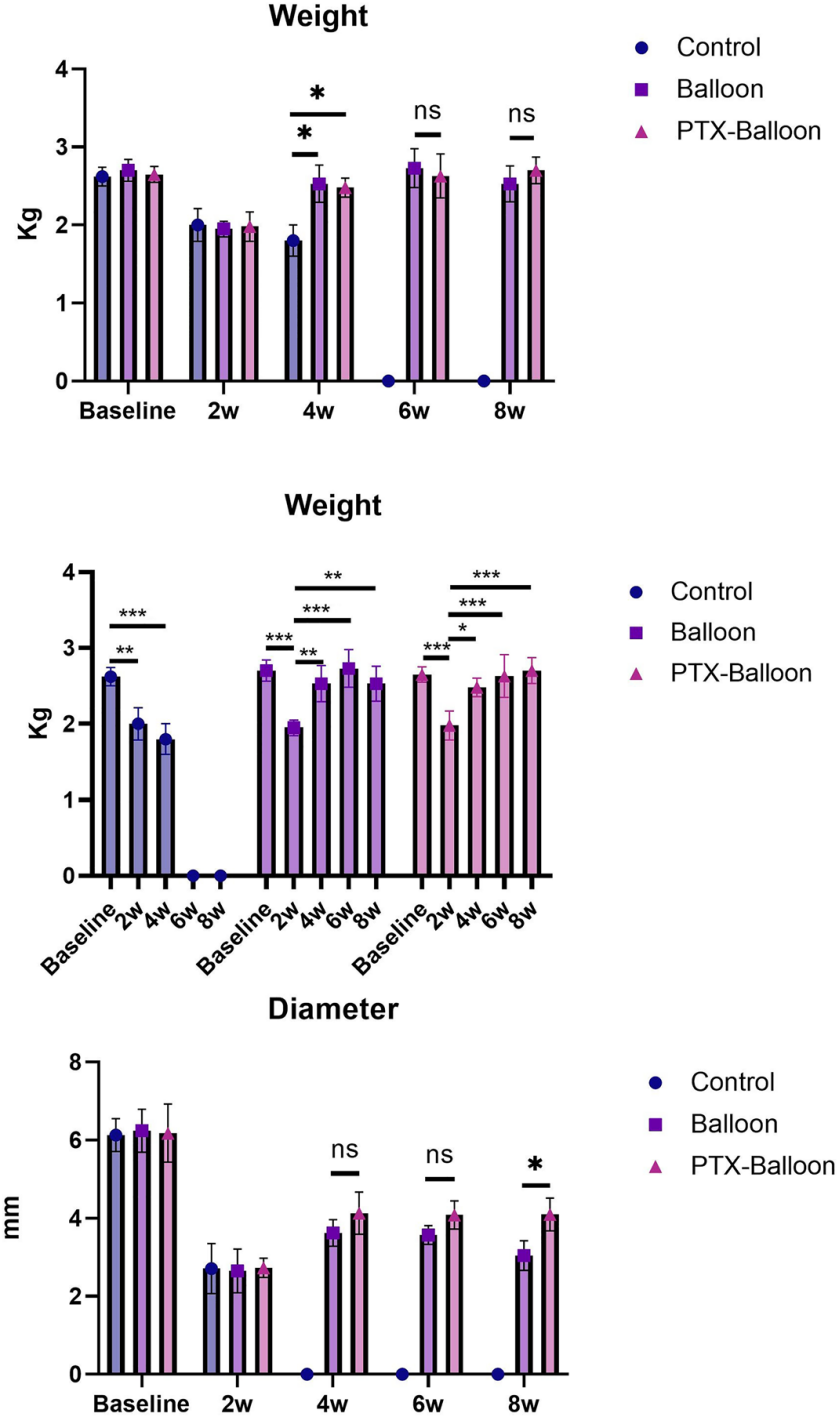
Analysis of weight and esophageal diameter. (**A**) Changes in the weights of rabbits in the 3 groups at different time points. (**B**) Changes in the weights of the rabbits in the 3 groups over time. (**C**) Changes in the esophageal diameter of rabbits in the 3 groups at different time points. P values < 0.05 (*), < 0.01 (**), and < 0.001 (***).

Two weeks after the operation, weight loss was observed in all 3 groups, and there was no difference between the groups. Four weeks after surgery, the weight of the rabbits in the control group continued to decrease. However, the weights of rabbits in the balloon group and PTX balloon group increased. There was significant difference between the control group and the balloon group (p = 0.01) and between the control group and the PTX balloon group (p = 0.01). There was no significant difference between the balloon group and the PTX balloon group at 4 w post-operation (p = 0.95) or at 6 w (p = 0.85) or 8 w (p = 0.64) (Fig. 4A). The weight of the mice in both the balloon group and the PTX balloon group increased over time after balloon dilation (Fig. 4B).

### Esophageal diameter changes

There was no difference in the esophageal diameter among the 3 groups at baseline or 2 weeks post-operation. There was no difference between the balloon group and the PTX balloon group at 4 w (p = 0.35) or 6 w (p = 0.35) after the operation. There was a significant difference between the balloon group and the PTX balloon group at 8 w post-operation (p = 0.02) (Fig. 4C).

### Pathological analysis

**Figure 5:**
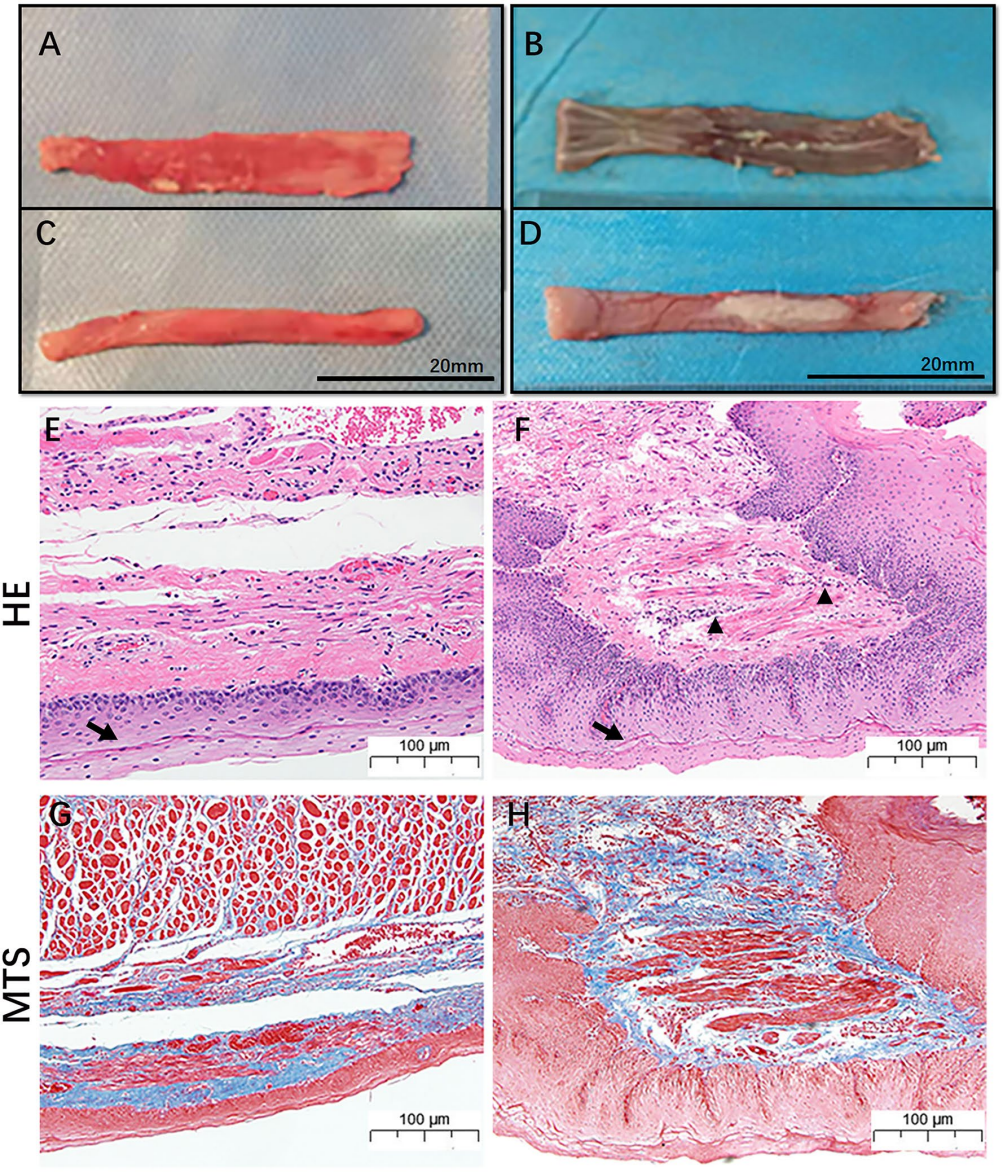
Representative general and pathological images of a normal esophagus and benign stenosis esophagus. (**A,C**) Inner and outer surfaces of the normal esophagus; (**B**,**D**) Inner and outer surfaces of the esophagus with benign stenosis; (**E**,**F)** Representative HE staining image of the normal esophagus and the esophagus with benign stenosis (the black arrow indicates the squamous epithelium; the black triangle indicates inflammatory cell infiltration). (**G**,**H**) Representative MTS staining image of a normal esophagus and an esophagus with benign stenosis; blue staining indicates collagen deposition.

Two weeks after the operation, the esophageal mucosa was partially destroyed, and the lumen became hard (Fig. 5A–D). Pathology revealed by HE (Fig. 5E,F) and MTS (Fig. 5G,H) staining indicated infiltration of inflammatory cells, thickening of the squamous epithelium and deposition of collagen fibers.

**Figure 6:**
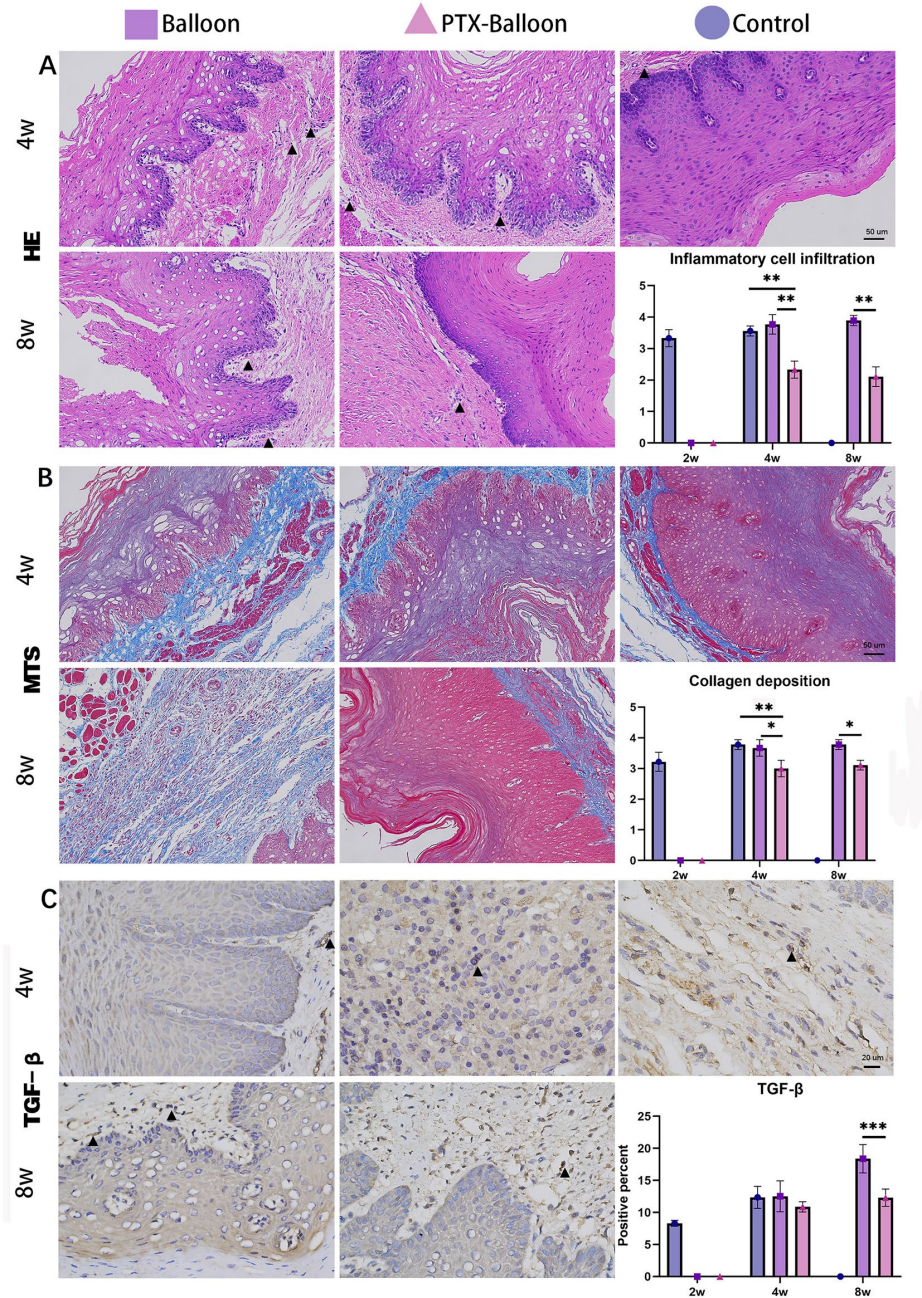
Pathological analysis of benign esophageal stenosis in the control group, balloon group, and PTX-coated balloon group at 4 w post-operation (2 w post-dilation) and 8 w post-operation (6 w post-dilation). (**A**) HE staining magnification, X200; scale bar = 50 µm (the black triangle indicates inflammatory cell infiltration); (**B**) MTS staining magnification, X200; scale bar = 50 µm (the blue staining indicates collagen deposition); (**C**) IHC staining at TGF-β magnification, X400; scale bar = 20 µm (the black triangle indicates positive staining). P values < 0.05 (*), < 0.01 (**), and < 0.001 (***).

Four weeks after surgery, the degree of inflammatory cell infiltration in the PTX balloon group was significantly lower than that in the control group (p = 0.002) and balloon group (p = 0.001). There was no difference between the control group and the balloon group (p = 0.60) (Fig. 6A). The degree of collagen deposition in the PTX balloon group was significantly lower than that in the control group (p = 0.002) and balloon group (p = 0.03). There was no difference between the control group and the balloon group (p = 0.84) (Fig. 6B). The results of TGF-β IHC indicated that there was no difference among the 3 groups (Fig. 6C).

Eight weeks after surgery, the percentage of cells positive for TGF-β (p < 0.001), the degree of inflammatory cell infiltration (p = 0.02) and the degree of collagen deposition (p = 0.02) in the PTX balloon group were significantly lower than those in the balloon group (Fig. 6A–C).

## Discussion

In this study, a rabbit model of benign esophageal stenosis was developed to explore the application of PTX-coated balloons. The main findings were as follows: First, 4% NaOH solution injection with balloon occlusion assistance was a feasible method for establishing an esophageal benign stenosis model. Second, infiltration of inflammatory cells, thickening of the squamous epithelium and deposition of collagen fibers were the main pathological findings of benign esophageal stenosis. Third, compared to balloon angioplasty, the application of PTX-coated balloons for esophageal benign stenosis alleviated the local inflammatory response and collagen deposition. Finally, PTX inhibited the expression of TGF-β during the healing process.

The New Zealand rabbit was chosen to establish an esophageal benign stenosis model for the following reasons. The rabbit esophagus is similar to the human infant esophagus in terms of structure and size. Second, the rabbit esophagus comprises three skeletal muscle layers: the inner and outer longitudinal layers and a middle circular layer. Third, rabbits are easier to handle and require more cost-effective maintenance than larger animals, such as pigs and dogs. At present, the most commonly used methods for constructing benign esophageal stenosis models include NaOH corrosion, endoscopic thermal injury, endoscopic mucosal resection, and submucosal dissection. Endoscopic mucosal resection, mucosal dissection and endoscopic thermal injury need to be completed under ultrafine esophageal endoscopy, which requires large amounts of experimental equipment and is difficult to perform^13–16^. In this study, a benign esophageal stenosis model was established by using balloon occlusion of the distal esophagus under DSA followed by catheter injection of NaOH solution. Balloon occlusion was able to control the duration and location of corrosion and prevent injury to the gastric cavity. In previous studies, different concentrations of NaOH solution were used to establish benign esophageal stenosis models; however, a low concentration of NaOH can lead to a low degree of esophageal stenosis and a long duration of development, while a high concentration of NaOH can easily lead to excessive esophageal injury, esophageal perforation and other serious complications^15,16^. In summary, in this study, a 4% concentration of NaOH was used to establish a benign esophageal stenosis model. The results showed that only 1 rabbit died of esophageal perforation, and the remaining rabbits all developed esophageal stenosis 2 weeks after the operation. These results suggest that it is feasible to establish an esophageal benign stenosis model with a 4% concentration of NaOH solution and balloon occlusion assistance under the guidance of DSA.

After injury to the esophageal mucosa, wound healing reactions occur at different stages. The healing process consists of the following three consecutive and partially overlapping stages: (1) the inflammatory reaction stage, which comprises neutrophils, macrophages and leukocyte infiltration; (2) the granulation tissue hyperplasia stage, which includes endothelial cell proliferation and fibrous proliferation; and (3) the maturation stage, which includes fibrocyte proliferation and type III collagen deposition^17^. In this study, a series of pathological manifestations, such as discontinuity of the esophageal mucosa, increased and thickened squamous epithelium, and fibrin deposition, were observed in the specimens.

Benign esophageal strictures are prone to relapse after balloon dilatation, and repeated dilatation is needed. The formation of scar tissue is an important process in recurrent esophageal stenosis^5^. In the process of balloon dilation, mechanical damage occurs to the wall of the esophagus and leads to a self-injury repair reaction, which in turn causes increased tissue tension and reduced elasticity at the site of esophageal dilation. Therefore, the esophagus cannot be effectively dilated during the process of swallowing food. This causes the recurrence of benign esophageal stenosis after esophagoplasty. Fibroblasts are the main cells responsible for the generation and proliferation of granulation tissue and scar tissue. Abnormal fibroblasts can promote fibrosis, resulting in excessive deposition of extracellular matrix^18^. Effectively inhibiting the proliferation of fibroblasts can play a key role in the treatment of benign esophageal stenosis.

PTX is an antiproliferative drug that acts on the microtubule/tubulin system, promoting the assembly of tubulin into microtubules and inhibiting the depolymerization of microtubules, which leads to the abnormal arrangement of microtubules and the formation of stellations, in turn resulting in the loss of the normal function of the spindle and the cessation of cell division in the G2 and M phases. The extensive application of PTX-coated balloons in coronary arteries and peripheral arteries has an inhibitory effect on endothelial cell proliferation. Previous studies have shown that PTX-coated stents can effectively reduce granulation tissue hyperplasia and scar formation^19^. This finding indicates the feasibility of the local application of PTX for the treatment of benign esophageal stenosis. Transforming growth factor (TGF) is a dimeric polypeptide growth factor that regulates cell activity and differentiation and plays an important role in the pathogenesis of various diseases. TGF-β is a major factor in the mechanism of mesenchymal cell synthesis in vitro and during tissue fibrosis^20,21^. In the study of Oldakovskiy^22^, losartan was used to prevent restenosis after balloon dilation in children with dystrophic epidermolysis bullosa (DEB). These findings demonstrated that losartan contributed to a decrease in restenosis frequency and an improvement in the nutritional status of children after balloon dilation. The main cause of DEB is a mutation in the type VII collagen gene. This increase in TGF-β activity promotes fibroblast transformation into myofibroblasts and leads to fibrosis. The antifibrotic effect of losartan is due to the suppression of TGF-β through an angiotensin II receptor type 1-mediated decrease in the expression of TGF-β activators such as thrombospondin 1. Taken together, our results showed that the expression of TGF-β was reduced in the PTX balloon group. These findings suggested that TGF-β plays a key role in the recurrence of benign stenosis after balloon dilation.

This study has several limitations. First, its small sample size may have affected the significance of the findings. However, these results can guide further, more detailed research. Second, the follow-up duration of 8 weeks may not have been enough to observe changes throughout the whole process, as was the case for patients with benign esophageal stenosis. Third, the mechanism of occurrence and recurrence of benign esophageal strictures was not studied in depth.

## Conclusion

In conclusion, the present study investigated the efficacy of PTX-coated balloons for treating benign esophageal stenosis. PTX-coated balloons can alleviate the local inflammatory response and collagen deposition when used during dilation treatment of benign esophageal stenosis.

## Data Availability

The datasets used and/or analysed during the current study available from the corresponding author on reasonable request.
